# The aqueous extract of *Albizia adianthifolia* leaves attenuates 6-hydroxydopamine-induced anxiety, depression and oxidative stress in rat amygdala

**DOI:** 10.1186/s12906-015-0912-0

**Published:** 2015-10-19

**Authors:** Galba Jean Beppe, Alain Bertrand Dongmo, Harquin Simplice Foyet, Théophile Dimo, Marius Mihasan, Lucian Hritcu

**Affiliations:** Laboratory of Animal Physiology, Faculty of Science, University of Yaoundé I, PO Box, 812, Yaoundé, Cameroon; Laboratory of Animal Biology and Physiology, University of Douala, PO Box, 24157, Douala, Cameroon; Department of Biological Sciences, Faculty of Science, University of Maroua, PO Box, 814, Maroua, Cameroon; Department of Biology, Alexandru Ioan Cuza University of Iasi, Bd. Carol I, No. 11, Iasi, Romania

**Keywords:** *Albizia adianthifolia* leaves extract, 6-hydroxydopamine-lesioned model, Anxiolytic-like effect, Antidepressant-like effect, Parkinson’s disease

## Abstract

**Background:**

While the *Albizia adianthifolia* (Schumach.) W. Wright (Fabaceae) is a traditional herb largely used in the African traditional medicine as analgesic, purgative, antiinflammatory, antioxidant, antimicrobial, memory-enhancer, anxiolytic and antidepressant drug, there are no scientific data that clarify the anxiolytic and antidepressant-like effects in 6-hydroxydopamine (6-OHDA)-lesioned animal model of Parkinson’s disease. This study was undertaken in order to identify the effects of aqueous extract of *A. adianthifolia* leaves on 6-hydroxydopamine-induced anxiety, depression and oxidative stress in the rat amygdala.

**Methods:**

The effect of the aqueous extract of *A. adianthifolia* leaves (150 and 300 mg/kg, orally, daily, for 21 days) on anxiety and depression was assessed using elevated plus-maze and forced swimming tests, as animal models of anxiety and depression. Also, the antioxidant activity in the rat amygdala was assessed using assessed using superoxide dismutase, glutathione peroxidase and catalase specific activities, the total content of the reduced glutathione, protein carbonyl and malondialdehyde levels. Statistical analyses were performed using by one-way analysis of variance (ANOVA). Significant differences were determined by Tukey’s *post hoc* test. *F* values for which *p* < 0.05 were regarded as statistically significant. Pearson’s correlation coefficient and regression analysis were used in order to evaluate the connection between behavioral measures, the antioxidant defence and lipid peroxidation.

**Results:**

6-OHDA-lesioned rats exhibited the following: decrease of the exploratory activity, the percentage of the time spent and the number of entries in the open arm within elevated plus-maze test and decrease of swimming time and increase of immobility time within forced swimming test. Administration of the aqueous extract significantly exhibited anxiolytic- and antidepressant-like effects and also antioxidant potential in the rat amygdala.

**Conclusions:**

Our results suggest that the aqueous extract ameliorates 6-OHDA-induced anxiety and depression by attenuation of the oxidative stress in the rat amygdala. These pieces of evidence accentuate its use in traditional medicine.

## Background

Parkinson’s disease (PD) is a neurodegenerative disorder characterized by a massive and progressive degeneration of the dopaminergic neurons in the substantia nigra (SN) [1]. About 30-40 % of people with PD experience a depressive disorder [2] and most have symptoms of depression at some stage [3]. Up to 40 % have symptoms of anxiety and around 30 % experience an anxiety disorder [4]. About 40 % of people with PD and depression also have an anxiety disorder. The link between PD, depression and anxiety is complex. Its neuropathology includes the degeneration of dopaminergic nigrostriatal pathway, which is a cumulative effect of glutathione depletion, iron deposition, increased lipid peroxidation, oxidative DNA damage, mitochondrial dysfunction, excitotoxicity and alterations in antioxidant enzymes activities [5, 6].

Previous studies indicated the role of the amygdala in regulating anxiety, depression responses and fear memory in both humans and animals [7]. Furthermore, the dopamine neurotransmitter system is involved in a large number of higher brain functions, and regulates the activity of the amygdala [8].

The most widely used animal models of PD involve intracranial infusion of the neurotoxin 6-OHDA directly into the ascending dopaminergic forebrain bundle, thereby inducing severe dopaminergic neuronal degeneration associated with profound deficits in feeding, drinking, sensorimotor and learning functions [9, 10].

Oxidative stress mechanism gets more prominent in the process of ageing, thus is the most important risk factor for developing PD [11]. Mitochondrial dysfunction along with damaged proteins and lipids due to oxidative stress plays a key role in PD pathological process [12].

*A. adianthifolia* is a medicinal plant widely distributed in Africa and in Cameroon. Aqueous and ethanol extracts of *A. adianthifolia* (stem bark) used in Southern Africa to treat memory loss and Alzheimer’s disease, have been screened for acetylcholinesterase inhibitory activity [13]. Kim et al. [14] reported that the aqueous extract of *A. julibrissin* had anxiolytic-like effects in rats as assessed using the elevated plus-maze test. Also, a recent study indicated that julibroside C1 extracted from *A. julibrissin* stem bark produced potent anxiolytic-like effects in mice [15]. Previously published data by our group demonstrated by HPLC analyses that among flavones, the main constituent of the aqueous extract of *A. adianthifolia* leaves is apigenin, responsible for the observed cognitive-enhancing effects in the 6-OHDA-lesion rodent model of PD [16]. Moreover, it was claimed that saponins from *Albizia lebbeck* could be used in treatment of Alzheimer’s and PD [17].

In this study we examined the effects of the aqueous extract of *A. adianthifolia* leaves on anxiety and depression levels, as well as the importance of the aqueous extract in oxidative stress status in the amygdala of 6-OHDA-treated rats. Correlation between the anxiety-depression-like behaviors and the levels of the main oxidative stress markers from the amygdala of 6-OHDA-treated rats, as a result of the aqueous extract administration was also investigated.

## Methods

### Plant material and plant extract

*Albizia adianthifolia* leaves were collected in Elounden, near Yaoundé (Cameroon) in June 2010 and identified by Dr. Nolé Tsabang at the National Herbarium, Yaoundé where a voucher specimen (N° HNC 29997) was registered and deposited for ready reference. All locations where the plant was collected were not privately-owned or protected in any way and the field studies did not involve endangered or protected species. *A. adianthifolia* leaves were air dried and pulverized into fine powder. Five hundred grams of pulverized sample material was macerated in 5 L of distilled water for 48 h at room temperature and shaking regularly. Then the mixture was filtered through Whatman filter paper no. 3. The aqueous extract was then lyophilized to obtain powder used for our various tests. The given powder yield was 4 % (v/w). The dried extract was dissolved in distilled water and administered by gastric gavage to animals at the doses of 150 and 300 mg/kg body weight.

### Animals

60 male Wistar rats weighing 350 ± 50 g at the start of the experiment were used. The animals were housed in a temperature and light-controlled room (22 °C, a 12-h cycle starting at 08:00 h) and were fed and allowed to drink water *ad libitum*. Rats were treated in accordance with the guidelines of animal bioethics from the Act on Animal Experimentation and Animal Health and Welfare from Romania and all procedures were in compliance with Directive 2010/63/EU of the European Parliament and of the Council of 22 September 2010 on the protection of animals used for scientific purposes. The protocol was approved by the Committee on the Ethics of Animal Experiments of the Alexandru Ioan Cuza University of Iasi, Romania (Permit Number: 1502723). All surgery was performed under sodium pentobarbital anesthesia, and all efforts were made to minimize animal suffering and to reduce the number of animal used. For the elevated plus-maze and forced swimming tests were employed the same group of rats.

### Drugs

6-Hydroxydopamine (free base, 6-OHDA), desipramine, sodium pentobarbital, diazepam hydrochloride and tramadol (Sigma-Aldrich, Germany) were dissolved in an isotonic solution (0.9 % NaCl). All other reagents were purchased from Sigma-Aldrich, Germany.

### Neurosurgery

All surgical procedures were conducted under aseptic conditions, under sodium pentobarbital (50 mg/kg b.w., i.p.,) anesthesia. Rats were mounted in the stereotaxic apparatus with the nose oriented 11° below horizontal zero plane. Thereafter, the animals received a right-unilaterally intranigral injections of 5 μL of 0.9 % saline containing 2.5 μg/μL 6-OHDA (free base) and 0.2 % ascorbic acid (w/v) at a rate of 1 μL/min at the following coordinates: 5.5 mm posterior to bregma; 2.0 mm lateral to the midline; 7.4 mm ventral to the surface of the cortex, according to the rat brain atlas [18]. All rats sustaining 6-OHDA lesion were pretreated with desipramine (25 mg/kg, i.p. in saline) 30 min before anesthesia in order to protect noradrenergic system. The rats in the control group (sham-operated) were given 5 μL of 0.9 % saline - 0.2 % ascorbic acid administered in a similar manner as the solution containing 6-OHDA.

### Postoperative care

Recovery from anesthesia took approximately 6 h. The rats were kept in well-ventilated room at 22 °C in individual cages till they gained full consciousness and were then housed together in a group of five animals per cage. Food was kept inside the cages during the first week so that animals could easily access it without any physical trauma due to surgical intervention.

### Drug administration

The rats were divided into 6 groups (10 per group): (1) control group (sham-operated, received orally distilled water treatment); (2) Diazepam alone-treated group (sham-operated, DZP, 1.5 mg/kg); (3) Tramadol alone-treated group (sham-operated, TRM, 10 mg/kg); (4) 6-OHDA-lesioned group, as negative control, received orally distilled water treatment; (5) 6-OHDA-lesioned group received orally 150 mg/kg of the aqueous extract of *A. adianthifolia* leaves treatment (6-OHDA + AE (150 mg/kg)) and (6) 6-OHDA-lesioned group received orally 300 mg/kg of the aqueous extract of *A. adianthifolia* leaves treatment (6-OHDA + AE (300 mg/kg)). The administration of the distilled water and the aqueous extract was performed orally by gastric gavage with rat biomedical needles (length 7.62 cm, ball diameter 4 mm, straight). The volume administered was 10 mL/kg of body weight, daily, for 21 consecutive days after neurosurgery. The aqueous extract doses (150 mg/kg and 300 mg/kg) used in this experiment were chosen since they have been demonstrated by our group to provide significant effects on memory formation and antioxidant profile [16]. Diazepam hydrochloride (Sigma-Aldrich, Germany) and tramadol (Sigma-Aldrich, Germany) were used as positive controls and were injected intraperitoneally (i.p.) in a volume of 1 mL/kg in the sham-operated rats, 1 h before behaviorally tested.

### Rotational behavior

The animals were tested for rotational behavior by pergolide (0.5 mg/kg, b.w., s.c., Sigma-Aldrich, Germany) 1 week after the 6-OHDA injection. The drug pergolide functions as a dopamine receptor agonist for D_2_ and D_1_ receptors and as a ligand to serotonin 5-HT_1A_, 5-HT_1B_, 5-HT_2A_, 5-HT_2B_, and 5-HT_2C_ receptors [19]. Also, it was argued that pergolide induced contralateral rotation in animals with striatal lesion after systemic administration [20]. Briefly, 1 min after pergolide injection, full rotations were counted in a cylindrical container (a diameter of 33 cm and a height of 35 cm) at 10 min intervals for 60 min in a quiet isolated room. Rotations in the ipsilateral and contralateral directions were counted separately and the analyses were based on the net scores (contralateral minus ipsilateral rotations) recorded for 60 min [21].

### Elevated plus-maze task

Behavior in the elevated plus-maze (EPM) is largely used to assess exploration, anxiety and motor behavior. The EPM consists of four arms, 49 cm long and 10 cm wide, elevated 50 cm above the ground. Two arms were enclosed by walls 30 cm high and the other two arms were exposed. The illumination was set at 400 lx. 30 min after the aqueous extract of *A. adianthifolia* leaves administration, each rat was placed in the center of the maze facing one closed arm. Behavior was observed for 5 min, and the time spent and number of entries into the open and enclosed arms was counted [22]. The percentages of time spent in the open arms (time spent in the open arms/time spent in all arms x 100) were calculated. In addition, the total number of open- and enclosed-arm entries, which indicates the locomotors activity of animals [23], was measured. An entry was defined as an animal placing all four paws into an arm, and no time was recorded when the animal was in the central area. The maze was cleaned with 10 % ethanol solution and dried with a cloth before the next animal was tested. Behavioral data were automatically tracked and recorded using ANY-maze behavioral software (Stoelting Co., USA, version 4.5).

### Forced swimming test (FST)

The possible antidepressant effects of the aqueous extract of *A. adianthifolia* leaves were assessed, basically using the same method described by Campos et al. [24] but with modification. On the first day of the experiments (pretest session), rats were individually placed into cylindrical recipients (diameter 30 cm, height 59 cm) containing 25 cm of water at 26 ± 1 °C. The animals were left to swim for 15 min before being removed, dried and returned to their cages. The procedure was repeated 24 h later, in a 6 min swim session (test session), 30 min after the aqueous extract of *A. adianthifolia* leaves administration. During the test session, the following behavioral responses were recorded: (1) immobility (time spent floating with the minimal movements to keep the head above the water) and (2) swimming (time spent with active swimming movements).

### Biochemical parameter assay

Three weeks after the administration of aqueous extract of *A. adianthifolia* leaves in 6-OHDA-lesioned animals groups, all rats were deeply anesthetized (using sodium pentobarbital, 100 mg/kg b.w., i.p., Sigma-Aldrich, Germany) and decapitated and whole brains were removed. Bilateral amygdala was carefully excised. Each of the amygdala samples were weighted and homogenized (1:10) with Potter Homogenizer coupled with Cole-Parmer Servodyne Mixer in ice-cold 0.1 M potassium phosphate buffer (pH 7.4), 1.15 % KCl. The homogenate was centrifuged (15 min at 960 x *g*) and the supernatant was used for assays of SOD, GPX, CAT specific activities, total content of reduced GSH, protein carbonyl and MDA levels.

### Determination of SOD activity

The activity of superoxide dismutase (SOD, EC 1.15.1.1) was assayed by monitoring its ability to inhibit the photochemical reduction of nitroblue tetrazolium (NBT). Each 1.5 mL reaction mixture contained 100 mM TRIS/HCl (pH 7.8), 75 mM NBT, 2 μM riboflavin, 6 mM EDTA, and 200 μL of supernatant. Monitoring the increase in absorbance at 560 nm followed the production of blue formazan. One unit of SOD is defined as the quantity required to inhibit the rate of NBT reduction by 50 % as previously described by Winterbourn et al. [25]. The enzyme activity is expressed as units/mg protein.

### Determination of GPX activity

Glutathione peroxidase (GPX, E.C. 1.11.1.9) activity was analyzed by a spectrophotometric assay. A reaction mixture consisting of 1 mL of 0.4 M phosphate buffer (pH 7.0) containing 0.4 mM EDTA, 1 mL of 5 mM NaN_3_, 1 mL of 4 mM glutathione (GSH), and 200 μL of supernatant was pre-incubated at 37 °C for 5 min. Then 1 mL of 4 mM H_2_O_2_ was added and incubated at 37 °C for further 5 min. The excess amount of GSH was quantified by the DTNB method as previously described by Sharma and Gupta [26]. One unit of GPX is defined as the amount of enzyme required to oxidize 1 nmol GSH/min. The enzyme activity is expressed as units/mg protein.

### Determination of CAT activity

Catalase (CAT, EC 1.11.1.6) activity was assayed following the method of Sinha [27]. The reaction mixture consisted of 150 μL phosphate buffer (0.01 M, pH 7.0), 100 μL supernatant. Reaction was started by adding 250 μL H_2_O_2_ 0.16 M, incubated at 37 °C for 1 min and reaction was stopped by addition of 1.0 mL of dichromate: acetic acid reagent. The tubes were immediately kept in a boiling water bath for 15 min and the green colour developed during the reaction was read at 570 nm on a spectrophotometer. Control tubes, devoid of enzyme, were also processed in parallel. The enzyme activity is expressed as μmol of H_2_O_2_ consumed/min/mg protein.

### Total content of reduced GSH

Glutathione (GSH) was measured following the method of Fukuzawa and Tokumura [28]. 200 μL of supernatant was added to 1.1 mL of 0.25 M sodium phosphate buffer (pH 7.4) followed by the addition of 130 μL DTNB 0.04 %. Finally, the mixture was brought to a final volume of 1.5 mL with distilled water and absorbance was read in a spectrophotometer at 412 nm and results were expressed as μg GSH/μg protein.

### Determination of protein carbonyl level

The extent of protein oxidation in the amygdala was assessed by measuring the content of protein carbonyl groups, using 2,4- dinitrophenylhydrazine (DNPH) derivatization as described by Oliver et al. [29] and following the indications of Luo and Wehr [30]. Basically, the supernatant fraction was divided into two equal aliquots containing approximately 2 mg of protein each. Both aliquots were precipitated with 10 % trichloroacetic acid (TCA, w/v, final concentration). One sample was treated with 2 N HCl, and the other sample was treated with an equal volume of 0.2 % (w/v) DNPH in 2 N HCI. Both samples were incubated at 25 °C and stirred at 5 min intervals. The samples were then reprecipitated with 10 % TCA (final concentration) and subsequently extracted with ethanol-ethyl acetate (1:1, v/v) and then reprecipitated at 10 % TCA. The pellets were carefully drained and dissolved in 6 M guanidine hydrochloride with 20 mM sodium phosphate buffer, pH 6.5. Insoluble debris was removed by centrifugation at 13 000 x *g* at 4 °C. The absorbance at 370 nm of the DNPH-treated sample versus the HCl control was recorded, and the results are expressed as nmols of DNPH incorporated/mg of protein based on an average absorptivity of 21.0 mM^−1^ cm^−1^ for most aliphatic hydrazones.

### Determination of MDA level

Malondialdehyde (MDA), which is an indicator of lipid peroxidation, was spectrophotometrically measured by using the thiobarbituric acid assay [31]. 200 μL of supernatant was added and briefly mixed with 1 mL of 50 % trichloroacetic acid in 0.1 M HCl and 1 mL of 26 mM thiobarbituric acid. After vortex mixing, samples were maintained at 95 °C for 20 min. Afterwards, samples were centrifuged at 960 x *g* for 10 min and supernatants were read at 532 nm. A calibration curve was constructed using MDA as standard and the results were expressed as nmol/mg protein.

### Estimation of protein concentration

Estimation of protein was done using a bicinchoninic acid (BCA) protein assay kit (Sigma-Aldrich, Germany). The BCA protein assay is a detergent-compatible formulation based on BCA for the colorimetric detection and quantification of total protein, as previously described by Smith et al. [32].

### DNA fragmentation

Total DNA was isolated from the amygdala samples using the phenol/chloroform method as previously described by Ausubel et al. [33]. 50 mg tissue sample was digested overnight at 37 °C in 0.6 mL digestion buffer (100 mM NaCl, 10 mM TRIS/HCl, 25 mM EDTA pH 8.00, 0.5 % SDS) containing 0.1 mg/mL proteinase K (Boehringer Mannheim, Germany). The digest was extracted with equal volumes of TRIS-saturated phenol (pH 8.0) (Roti-phenol, Roth, Germany) by shaking gently to completely mix the two phases. The phases were then separated by centrifugation and the aqueous phase (approx. 0.6 ml) was transferred to another tube avoiding interphase. The DNA was then precipitated by adding 300 μL of 7.5 M ammonium acetate (i.e., 1/2 of volume) and equal volume of 100 % ethanol at room temperature and shaken gently to mix thoroughly. DNA seen as stringy precipitate was pelleted by centrifugation and washed with 70 % ethanol to remove traces of sodium dodecyl sulfate and phenol. After removing ethanol, DNA was air-dried for 10 min at room temperature and suspended with 50 μL of 10 mM TRIS (pH 8.0), 1 mM EDTA. DNA content was determined spectrophotometrically by absorbance at 260 nm and the purity of the DNA was confirmed by a ratio >1.8 at 260/280 nm. Approximately 0.5 mg genomic DNA was dissolved in a mixture of 10 μL of TRIS-EDTA and 5 μL of gel loading buffer (0.25 % bromophenol blue, 0.25 % xylene cyanol FF, 30 % (v/v) glycerol) and then loaded on a 1.5 % agarose gel in TRIS-boric acid-EDTA (TBE) buffer (89 mM Tris boric acid, 2 mM EDTA, pH 8.0). Electrophoresis was performed in TBE at 120 V until sufficient resolution was obtained. A 1-kb DNA ladder (New England Biolabs, Ipswich, MA) was used as a standard size marker. The bands were visualized by ethidium bromide staining under UV light.

### 6-OHDA-lesion control

Pergolide-induced rotational behavior test was employed to validate unilateral damage to dopamine nigrostriatal neurons. The pergolide results confirm the impairment of the dopaminergic system as previously described [16].

### Statistical analysis

Behavioral scores within elevated plus-maze and forced swimming tests and biochemical data were analyzed by one-way analysis of variance (ANOVA) using XLSTAT version 2012. 1.01 software, Addinsoft. All results are expressed as mean ± S.E.M. *F* values for which *p* < 0.05 were regarded as statistically significant. Significant differences were determined by Tukey’s *post hoc* test. Pearson’s correlation coefficient and regression analysis were used in order to evaluate the connection between behavioral measures, the antioxidant defence and lipid peroxidation.

## Results

### Effect pergolide on rotational behavior

**Fig. 1:**
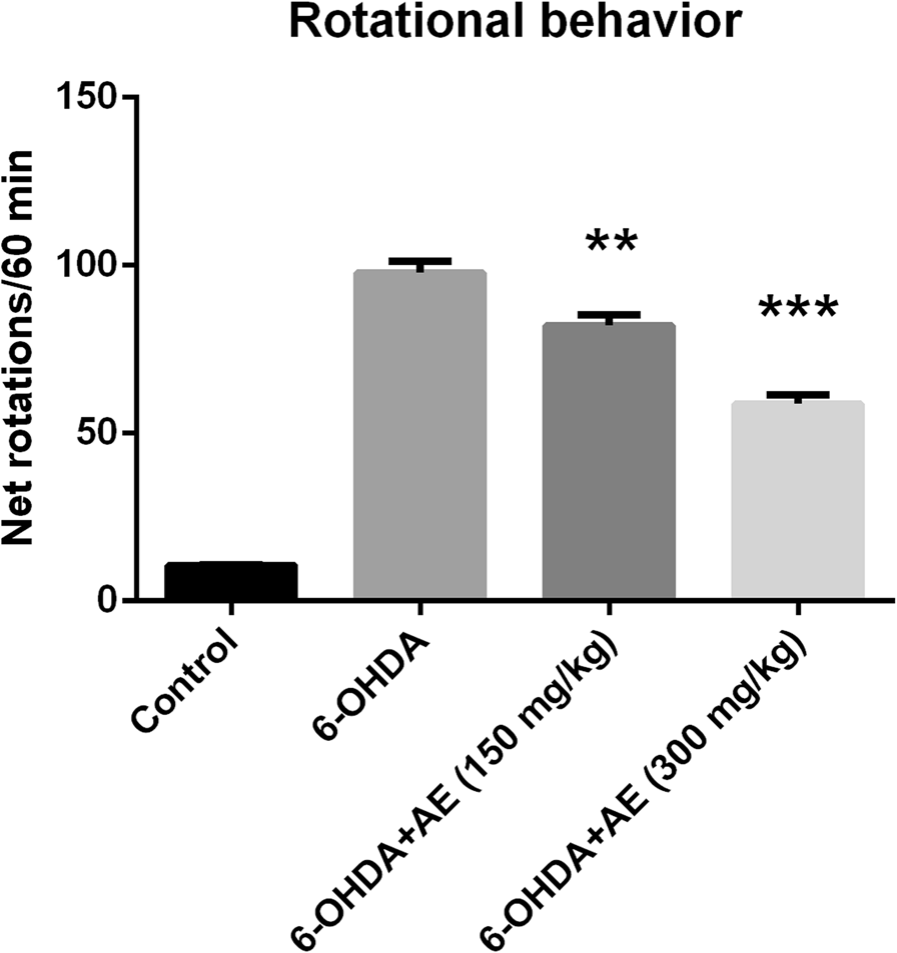
Protective effects of the aqueous extract of *Albizia adianthifolia* leaves (150 and 300 mg/kg) on pergolide (0.5 mg/kg, b.w., s.c.)-induced rotational behavior in 6-OHDA-lesioned rats. 1 week after 6-OHDA injection, the number of net rotation contralateral to the lesion side was increased. However, the administration of the aqueous extract restored the decrease in the number of net rotation. Values are means ± S.E.M. (*n* = 10 animals per group), **p < 0.001, ***p < 0.0001 vs. 6-OHDA alone treated-group

Pergolide-induced rotational behavior was analyzed to assess the unilateral degeneration of the dopamine nigrostriatal neurons. Control group did not show any significant bias in turning behavior after receiving pergolide injection. In contrast, rats exhibited contralateral rotational behavior following pergolide challenge in 1 week after the unilateral administration of 6-OHDA into the SN. In the rotational behavioral test, ANOVA revealed an attenuation of asymmetric motor behavior (F(3,36) = 20.03, *p* < 0.0001) in experimental animals treated with the aqueous extract of *A. adianthifolia* leaves (150 and 300 mg/kg) as compared to control group (Fig. 1). Additionally, Tukey’s *post hoc* analysis revealed a significant difference between the control and 6-OHDA groups (*p* < 0.0001), control and 6-OHDA + AE (150 mg/kg) groups (*p* < 0.0001), control and 6-OHDA + AE (300 mg/kg) groups (*p* < 0.0001), 6-OHDA and 6-OHDA + AE (150 mg/kg) groups (*p* < 0.001), 6-OHDA and 6-OHDA + AE (300 mg/kg) groups (*p* < 0.0001) and 6-OHDA + AE (150 mg/kg) and 6-OHDA + AE (300 mg/kg) groups (*p* < 0.0001) (Fig. 1).

### Effect of *Albizia adianthifolia* extract on elevated plus-maze behavior

**Fig. 2:**
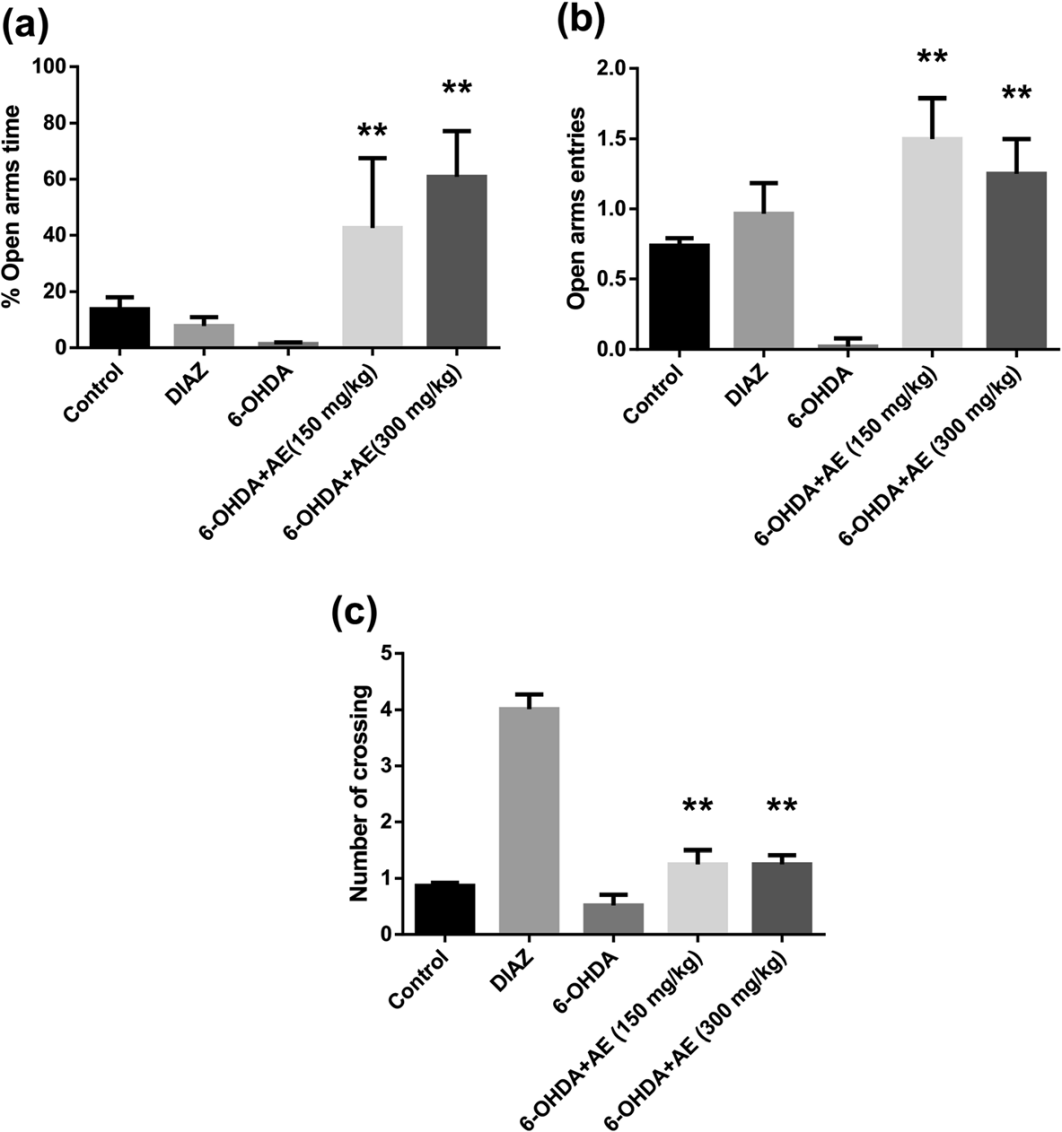
Effects of the aqueous extract of *Albizia adianthifolia* leaves (150 and 300 mg/kg) in the elevated plus-maze test on **a** the percentage of the time spent in the open arms, **b** the number of open-arm entries and **c** the number of crossing in the 6-OHDA-treated rats. Values are means ± S.E.M. (*n* = 10 animals per group), **p < 0.01, vs. 6-OHDA alone treated-group

As can be seen in Fig. 2a, in the elevated plus-maze ANOVA revealed a significant increase of the percentage of the time spent in the open arms in 6-OHDA-lesioned groups treated with low-and high-doses (150 and 300 mg/kg) of the aqueous extract of *A. adianthifolia* leaves (F(3,36) = 3.51, *p* < 0.05), suggesting significant anxiolytic effects. Additionally, Tukey’s *post hoc* analysis revealed a significant difference between the control and 6-OHDA groups (*p* < 0.05), control and 6-OHDA + AE (150 mg/kg) groups (*p* < 0.05), control and 6-OHDA + AE (300 mg/kg) groups (*p* < 0.01), DIAZ and 6-OHDA groups (*p* < 0.05), DIAZ and 6-OHDA + AE (150 mg/kg) groups (*p* < 0.001), DIAZ and 6-OHDA + AE (300 mg/kg) (*p* < 0.001), 6-OHDA and 6-OHDA + AE (150 mg/kg) groups (*p* < 0.01) and 6-OHDA and 6-OHDA + AE (300 mg/kg) groups (*p* < 0.01) (Fig. 2a) for the percentage of the time spent in the open arms, indicating that the aqueous extract of *A. adianthifolia* leaves has anxiolytic-like profile. Non-significant differences between control and DIAZ were observed (*p* > 0.05).

As can be seen in Fig. 2b, in the elevated plus-maze ANOVA revealed a significant increase of the number of open-arm entries in 6-OHDA-lesioned groups treated with low-and high-doses (150 and 300 mg/kg) of the aqueous extract of *A. adianthifolia* leaves (F(3,36) = 8.03, *p* < 0.001), suggesting significant anxiolytic effects. Additionally, Tukey’s *post hoc* analysis revealed a significant difference between the control and 6-OHDA groups (*p* < 0.001), control and 6-OHDA + AE (150 mg/kg) groups (*p* < 0.05), control and 6-OHDA + AE (300 mg/kg) groups (*p* < 0.01), DIAZ and 6-OHDA groups (*p* < 0.05), 6-OHDA and 6-OHDA + AE (150 mg/kg) groups (*p* < 0.0001) and 6-OHDA and 6-OHDA + AE (300 mg/kg) groups (*p* < 0.001) (Fig. 2b) for the number of open-arm entries, indicating that the aqueous extract of *A. adianthifolia* leaves has anxiolytic-like profile as suggested above. Non-significant differences between control and DIAZ were observed (*p* > 0.05).

As can be seen in Fig. 2c, in the elevated plus-maze ANOVA revealed a significant increase on the exploratory locomotor activity in 6-OHDA-lesioned groups treated with low-and high-doses (150 and 300 mg/kg) of the aqueous extract of *A. adianthifolia* leaves (F(3,36) = 48.87, *p* < 0.0001), as evidenced by the number of crossing. Additionally, Tukey’s *post hoc* analysis revealed a significant difference between the control and DIAZ groups (*p* < 0.0001), control and 6-OHDA groups (*p* < 0.05), control and 6-OHDA + AE (150 mg/kg) groups (*p* < 0.01), control and 6-OHDA + AE (300 mg/kg) groups (*p* < 0.01), DIAZ and 6-OHDA groups (*p* < 0.0001), DIAZ and 6-OHDA + AE (150 mg/kg) groups (*p* < 0.0001), DIAZ and 6-OHDA + AE (300 mg/kg) groups (*p* < 0.0001), 6-OHDA and 6-OHDA + AE (150 mg/kg) groups (*p* < 0.01) and 6-OHDA and 6-OHDA + AE (300 mg/kg) groups (*p* < 0.01) (Fig. 2c) for the number of crossing. Also, significant differences between control and DIAZ were observed (*p* < 0.0001).

### Effect of *Albizia adianthifolia* extract in the rat forced swimming test

**Fig. 3:**
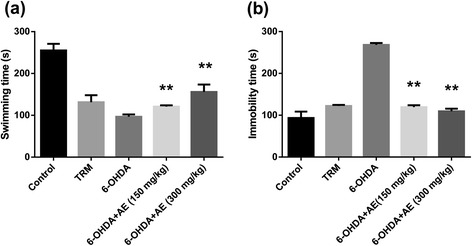
Effects of the aqueous extract of *Albizia adianthifolia* leaves (150 and 300 mg/kg) on **a** swimming time and **b** immobility time in the 6-OHDA-treated rats during the 6 min period in the forced swimming test. Values are means ± S.E.M. (*n* = 10 animals per group), **p < 0.01 vs. 6-OHDA alone treated-group

In the forced swimming test, ANOVA revealed a significant effect on depressive-like response in 6-OHDA-lesioned groups treated with low-and high-doses (150 and 300 mg/kg) of the aqueous extract of *A. adianthifolia* leaves, as evidenced by the swimming time (F(3,36) = 21.09, *p* < 0.0001) (Fig. 3a) and the immobility time (F(3,36) = 78.59, *p* < 0.0001) (Fig. 3b). Additionally, Tukey’s *post hoc* analysis revealed significant differences between the control and TRM groups (*p* < 0.0001), control and 6-OHDA groups (*p* < 0.0001), control and 6-OHDA + AE (150 mg/kg) groups (*p* < 0.0001), control and 6-OHDA + AE (300 mg/kg) groups (*p* < 0.0001), TRM and 6-OHDA groups (*p* < 0.05), 6-OHDA and 6-OHDA + AE (150 mg/kg) groups (*p* < 0.01), 6-OHDA and 6-OHDA + AE (300 mg/kg) groups (*p* < 0.01) and 6-OHDA + AE (150 mg/kg) and 6-OHDA + AE (300 mg/kg) groups (*p* < 0.01) for the swimming time (Fig. 3a).

Moreover, Tukey’s *post hoc* analysis revealed significant differences between the control and TRM groups (*p* < 0.01), control and 6-OHDA groups (*p* < 0.0001), TRM and 6-OHDA groups (*p* < 0.0001), 6-OHDA and 6-OHDA + AE (150 mg/kg) groups (*p* < 0.0001) and 6-OHDA and 6-OHDA + AE (300 mg/kg) groups (*p* < 0.0001) for the immobility time (Fig. 3b).

### Effect of *Albizia adianthifolia* extract on SOD, GPX and CAT activities

**Fig. 4:**
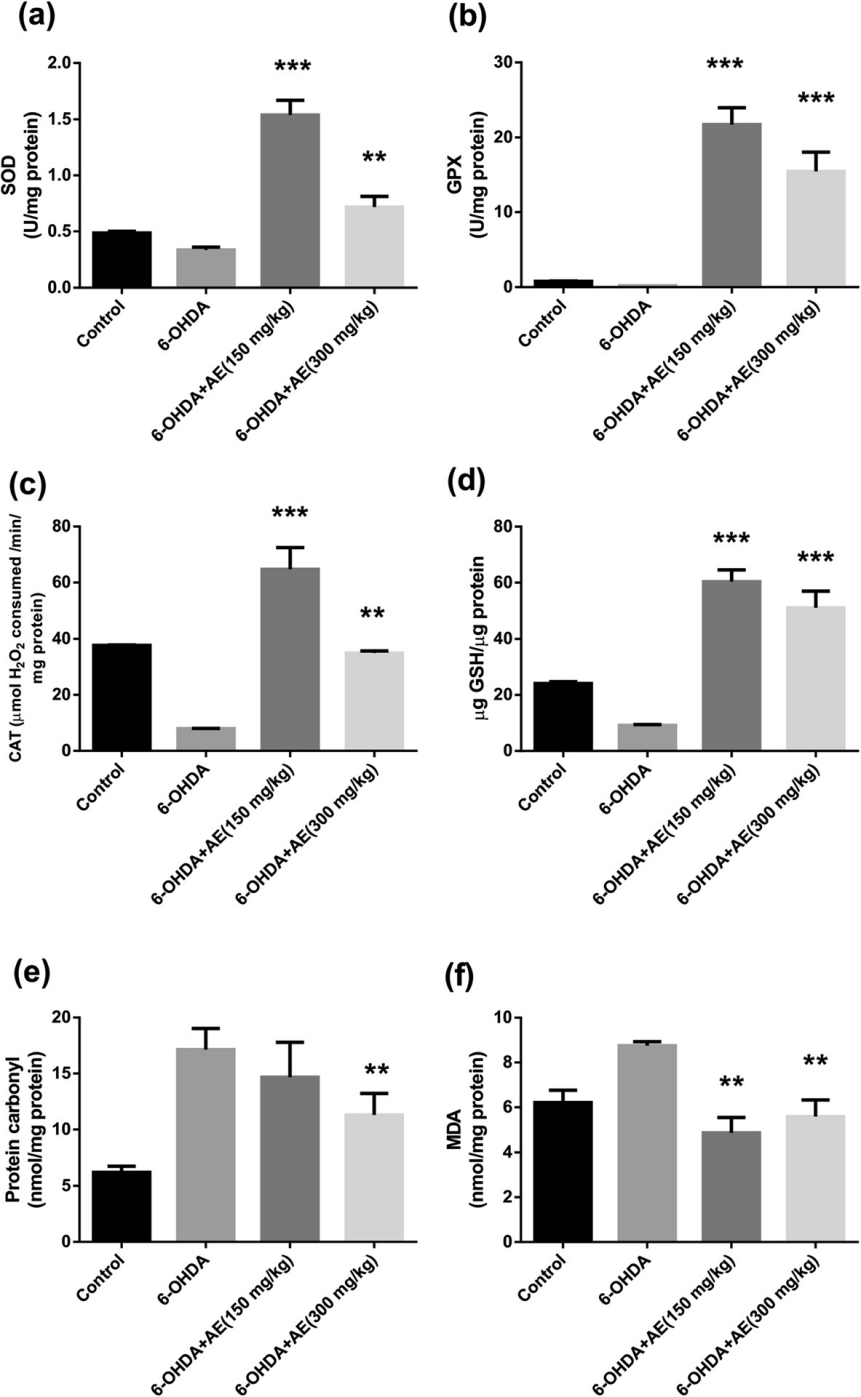
Effects of the aqueous extract of *Albizia adianthifolia* leaves (150 and 300 mg/kg) on **a** SOD, **b** GPX and **c** CAT specific activities, on **d** total content of reduced GSH, **e** protein carbonyl and **f** MDA levels in the 6-OHDA-treated rats. Values are means ± S.E.M. (*n* = 10 animals per group), **p < 0.001, ***p < 0.0001 vs. 6-OHDA alone treated-group

For SOD specific activity estimated in the rat amygdala homogenates, ANOVA revealed a significant overall effect (F(3,36) = 42.68, *p* < 0.0001) (Fig. 4a). Additionally, Tukey’s *post hoc* analysis revealed significant differences between the control and 6-OHDA groups (*p* < 0.01), control and 6-OHDA + AE (150 mg/kg) groups (*p* < 0.0001), control and 6-OHDA + AE (300 mg/kg) groups (*p* < 0.01), 6-OHDA and 6-OHDA + AE (150 mg/kg) groups (*p* < 0.0001), 6-OHDA and 6-OHDA + AE (300 mg/kg) groups (*p* < 0.001) and 6-OHDA + AE (150 mg/kg) and 6-OHDA + AE (300 mg/kg) groups (*p* < 0.0001) for SOD specific activity.

For GPX specific activity estimated in the rat amygdala homogenates, ANOVA revealed a significant overall effect (F(3, 36) = 40.20, *p* < 0.0001) (Fig. 4b). Additionally, Tukey’s *post hoc* analysis revealed significant differences between the control and 6-OHDA + AE (150 mg/kg) groups (*p* < 0.0001), control and 6-OHDA + AE (300 mg/kg) groups (*p* < 0.001), 6-OHDA and 6-OHDA + AE (150 mg/kg) groups (*p* < 0.0001) and 6-OHDA and 6-OHDA + AE (300 mg/kg) groups (*p* < 0.001) for GPX specific activity.

For CAT specific activity estimated in the rat amygdala homogenates, ANOVA revealed a significant overall effect (F(3, 36) = 35.40, *p* < 0.0001) (Fig. 4c). Additionally, Tukey’s *post hoc* analysis revealed significant differences between the control and 6-OHDA groups (*p* < 0.001), control and 6-OHDA + AE (150 mg/kg) groups (*p* < 0.001), 6-OHDA and 6-OHDA + AE (150 mg/kg) groups (*p* < 0.0001), 6-OHDA and 6-OHDA + AE (300 mg/kg) groups (*p* < 0.001) and 6-OHDA + AE (150 mg/kg) and 6-OHDA + AE (300 mg/kg) groups (*p* < 0.001) for CAT specific activity.

### Effect of *Albizia adianthifolia* extract on total content of reduced GSH, protein carbonyl and MDA levels

For the total content of reduced GSH estimated in the rat amygdala homogenates, ANOVA revealed a significant overall effect (F(3, 36) = 41.79, *p* < 0.0001) (Fig. 4d). Additionally, Tukey’s *post hoc* analysis revealed significant differences between the control and 6-OHDA groups (*p* < 0.01), control and 6-OHDA + AE (150 mg/kg) groups (*p* < 0.0001), control and 6-OHDA + AE (300 mg/kg) groups (*p* < 0.001), 6-OHDA and 6-OHDA + AE (150 mg/kg) groups (*p* < 0.0001) and 6-OHDA vs. 6-OHDA + AE (300 mg/kg) groups (*p* < 0.0001) for the total content of reduced GSH.

For the protein carbonyl level ANOVA revealed a significant overall effect (F(3, 36) = 5.19, *p* < 0.01) (Fig. 4e). Additionally, Tukey’s *post hoc* analysis revealed significant differences between the control and 6-OHDA groups (*p* < 0.01), control and 6-OHDA + AE (150 mg/kg) groups (*p* < 0.01), control and 6-OHDA + AE (300 mg/kg) groups (*p* < 0.01) and 6-OHDA and 6-OHDA + AE (300 mg/kg) groups (*p* < 0.001) for protein carbonyl level.

For the MDA level ANOVA revealed a significant overall effect (F(3, 36) = 8.48, *p* < 0.001) (Fig. 4f). Additionally, Tukey’s *post hoc* analysis revealed significant differences between the control and 6-OHDA groups (*p* < 0.001), 6-OHDA and 6-OHDA + AE (150 mg/kg) groups (*p* < 0.001) and 6-OHDA and 6-OHDA + AE (300 mg/kg) groups (*p* < 0.01) for MDA level.

These results support the hypothesis that the aqueous extract of *A. adianthifolia* leaves may have induced a decrease in neuronal oxidative stress.

**Fig. 5:**
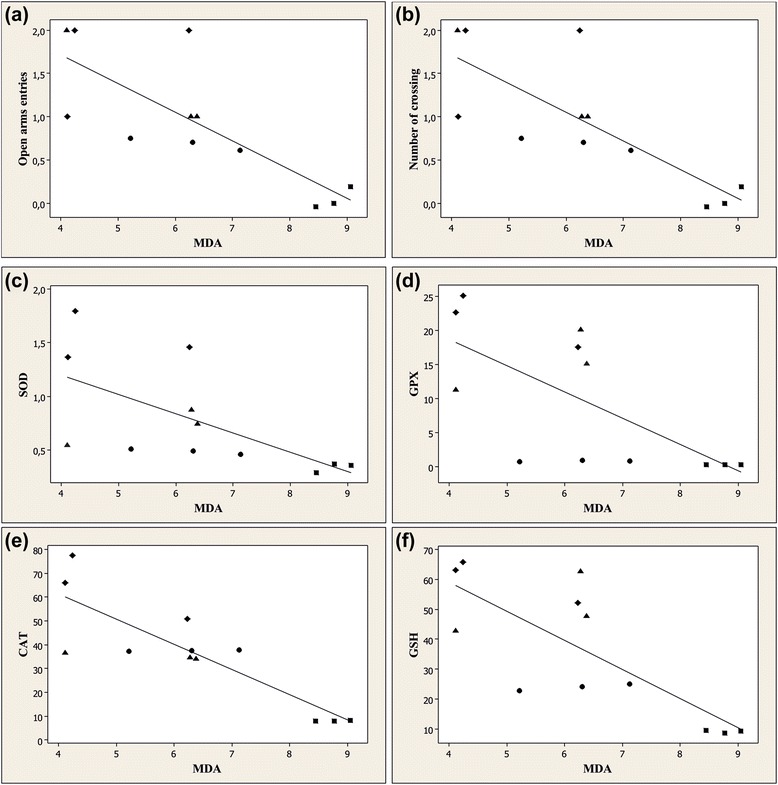
Pearson’s correlation between **a** the number of entries in the open arms vs. MDA, **b** the number of crossing vs. MDA, **c** SOD vs. MDA, **d** GPX vs. MDA, **e** CAT vs. MDA and **f** GSH vs. MDA in control group (●), 6-OHDA alone treated-group (■), 6-OHDA + AE (150 mg/kg) group (♦) and 6-OHDA + AE (300 mg/kg) group (▲)

More importantly, when linear regression was determined, significant correlations between the number of entries in the open arms vs. MDA (*n* = 40, *r* = −0.791, *p* < 0.001) (Fig. 5a), the number of crossing vs. MDA (*n* = 40, *r* = −0.872, *p* < 0.001) (Fig. 5b) were evidenced. Additionally, a significant correlation was evidenced by determination of the linear regression between SOD vs. MDA (*n* = 40, *r* = −0.632, *p* < 0.01) (Fig. 5c), GPX vs. MDA (*n* = 40, *r* = −0.675, *p* < 0.01) (Fig. 5d), CAT vs. MDA (*n* = 40, *r* = −0.855, *p* < 0.0001) (Fig. 5e) and GSH vs. MDA (*n* = 40, *r* = −0.771, *p* < 0.0001) (Fig. 5f).

These data suggest that anxiolytic responses within elevated plus-maze a and increase of the antioxidant defence along with decrease of lipid peroxidation could related with the involvement of the aqueous extract of *A. adianthifolia* leaves in neuroprotection against 6-OHDA-induced neuronal oxidative stress generation.

### Effect of *Albizia adianthifolia* extract on DNA fragmentation

**Fig. 6:**
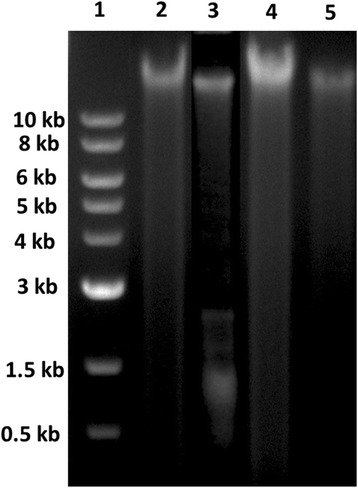
Effects of the aqueous extract of *Albizia adianthifolia* leaves (150 and 300 mg/kg) DNA fragmentation by agarose (1.5 %) gel electrophoresis. Lane 1: DNA ladder; lane 2: control group; lane 3: 6-OHDA alone treated-group; lane 4: 6-OHDA + AE (150 mg/kg) group and lane 5: 6-OHDA + AE (300 mg/kg) group

In our study, DNA cleavage patterns were absent in the aqueous extract groups as compared to 6-OHDA alone-treated group (Fig. 6), suggesting that the aqueous extract of *A. adianthifolia* leaves do not induce neurotoxicity and this effect could be related to its antioxidant activity.

## Discussion

The present study was aimed to examine the anxiety-like behavior and depressive-like response following administration of the aqueous extract of *A. adianthifolia* leaves in rats subjected to intranigral injection of 6-OHDA. It has been reported that 6-OHDA-induce lesion of the striatum causes depression- and anxiety-like behavior [34].

Previously, we have reported apigenin as the main component of the aqueous extract of *A. adianthifolia* leaves [16]. Several studies indicated anxiolytic and antidepressant activity of apigenin in rats exposed to behavioral tests [35]. Moreover, it is reported that apigenin modulates the γ-aminobutyric acid (GABA)ergic system to produce the biological effect .

PD is accompanied by non-motor symptoms, including olfactory loss, cognitive decline, depression and anxiety, which frequently appear in the early stages or even during the premotor phase of the disease [34]. Psychiatric symptoms have been related to monoaminergic deficits within components of the limbic system implicated in emotional and affective functions. In fact, the progressive death of midbrain dopaminergic neurons in PD is paralleled by the concomitant degeneration of noradrenergic and serotonergic systems [36].

The 6-OHDA lesion used in this study affects both dopamine and noradrenaline innervation [34]. Therefore, to assess the contribution of these systems to anxiety and depression conditions, we limited the lesion with 6-OHDA to the dopamine neurons by pre-treating the rats with the selective noradrenaline reuptake inhibitor, desipramine. In addition, the pergolide results confirm the impairment of the dopaminergic system.

Our data show that intranigral unilateral injection of 6-OHDA significantly decreased the percentage of the time spent in the open arms and the number of open-arm entries in the elevated plus maze test, two indicative parameters of anxiety. This indicates that the 6-OHDA-treated rats experienced high levels of anxiety and were suitable for evaluating the presumed anxiolytic substances. Notably, we found that administration of the aqueous extract (150 mg/kg and 300 mg/kg) counteracts anxiety of rats produced by the 6-OHDA lesion, suggesting potent anxiolytic effects. These results are strengthened by the fact that the benzodiazepine diazepam (DZP), well-known as positive standard anxiolytic [37], was used as positive control comparatively to the aqueous extract in all of our experimental conditions. DZP produced significant increases of the number of open-arm entries and in the number of crossing, while the percentage of time spent in the open arms was decreased as compared to control group. These data are consistent with the results of numerous previous studies, which have shown that DZP and other benzodiazepines produce significant anxiolytic effects in a variety of anxiolytic screening procedures, including elevated plus‐maze test procedures [38]. The pharmacological action of diazepam enhances the effect of the neurotransmitter GABA by binding to the benzodiazepine site on the GABA_A_ receptor (via the constituent chlorine atom) leading to central nervous system (CNS) depression. The anxiety indicators in the elevated plus-maze (the percentage of the time spent in the open arms and the number of open-arm entries) showed up being sensitive to the agents which were thought to act via the GABA_A_ receptor complex [39].

The anxiolytic-like effects of the aqueous extract could be explained by the presence of apigenin in large amounts, as previously evidenced by HPLC analysis [16]. However, it is suggested that apigenin has a clear anxiolytic-like effect in mice, without evidencing sedation or muscle relaxation effects. This effect could be mediated by the action of GABA receptor complex [40]. In agreement with these reports, our high-apigenin containing aqueous extract has increased anxiolytic-like behavior and exploratory activity in 6-OHDA-treated rats.

The forced swimming test has been validated as a suitable tool for predicting the antidepressant properties of drugs [41]. When rodents are forced to swim in a confined space, after an initial period of struggling, they would become immobile, resembling a state of despair and mental depression. This inescapable stressful situation can be evaluated by assessing different behavioral strategies [42].

Our data suggest that 6-OHDA increased the immobility time in the forced swimming test, a standard behavioral paradigm indicative of depression [34]. These findings are in line with a previous study performed in the 6-OHDA-lesioned rat [43]. Moreover, we found that treatment with the aqueous extract (150 mg/kg and 300 mg/kg) effectively reverted the increase of the immobility time and prolonged the swimming time displayed by the 6-OHDA-lesioned rats. We used tramadol as a positive control in the aqueous extract assay for all of our experimental conditions. Tramadol has been studied in the forced swimming test in rats, a test developed to predict the antidepressant action of drugs [44]. Tramadol is a unique drug with multiple modes of action. It is a weak agonist of the μ-opioid receptor but it also inhibits the reuptake of serotonin as well as norepinephrine. It is an analgesic and it is also considered as an antidepressant [45]. Also, tramadol exhibited significant effects on the immobility time while swimming time was decreased as compared to control group.

Oxidative stress enhancement also plays a pivotal role in 6-OHDA-neurotoxicity in experimental models of PD [46]. There is increasing evidence that oxidative stress due to increased generation of reactive oxygen species is strongly involved in the pathogenesis of PD, suggesting that pharmacological targeting of the antioxidant machinery may be of benefit in PD therapy [47]. Since 6-OHDA leads to the production of both H_2_O_2_ and superoxide radicals, decreasing of SOD activity leads to 6-OHDA-induced toxicity. Similarly, a decrease of GPX activity may be a cause of a decrease in the levels of GSH, because 6-OHDA induced cytotoxicity via H_2_O_2_ intracellular production which can be transformed in reactive hydroxyl radicals and produce cell damage [48]. The aqueous extract of *A. adianthifolia* leaves in the two doses used, reestablished the specific activities of SOD, GPX and CAT, increased GSH level along with decreased lipid peroxidation (MDA level) and protein oxidation (protein carbonyl level) in the amygdala homogenates. The increased activities of SOD, GPX, CAT, GSH level and the decreased levels of protein carbonyl and MDA induced by administration of the aqueous extract of *A. adianthifolia* leaves, implying that this plant extract possesses strong antioxidant property. The antioxidants SOD and GSH have a certain anti-depressant effect. Thus, anti-oxidation features in organisms are considered one way to resist depression [49]. The antioxidant effect observed could be attributed to apigenin the only flavonoid that was quantified [16]. Our finding are in line with other studies in which apigenin strongly scavenged the ROS overproduction and provided sufficient antioxidant effect by improving the antioxidative enzymes activity [11]. Also, apigenin was found to exert a variety of pharmacological actions on the central nervous system, such as anxiolytic and sedative properties [50]. Therefore, the observed effects of our plant extract could be attributed at least partially to apigenin.

Additionally, when linear regression was determined, a significant correlation between the number of entries in the open arms vs. MDA, the number of crossing vs. MDA, SOD vs. MDA, GPX vs. MDA, CAT vs. MDA and GSH vs. MDA was observed. These results may suggest that the increase of anxiolytic responses within elevated plus-maze and the antioxidant defence along with decrease of lipid peroxidation could be correlated with involvement of the aqueous extract in neuroprotection against 6-OHDA-induced oxidative stress generation in the rat amygdala. Also, we reported the absence of DNA cleavage patterns in the amygdala of the 6-OHDA-treated rats after administration of the aqueous extract, suggesting that the aqueous extract doesn’t possesses neurotoxic effects.

Regarding the limitation of our study we can add that there is an indirect behavioral evidence (e.g. rotational behavior) that the lesion worked rather than neurochemistry of the substantia nigra to confirm the size of lesion.

## Conclusions

In summary, the aqueous extract of *A. adianthifolia* leaves has anxiolytic and antidepressant effects, and may confer neuroprotection due to alleviation of oxidative stress in the rat amygdala induced by 6-OHDA injection. Furthermore, administration of the aqueous extract might offer a useful alternative or complementary choice in either the prevention or the treatment of psychiatric condition with relevance for PD.
